# Glycyrrhetinic acid protects against *Multidrug-resistant Acinetobacter baumannii*-induced lung epithelial cells injury by regulating inflammation and oxidative stress

**DOI:** 10.1186/s40360-023-00648-z

**Published:** 2023-01-30

**Authors:** Piaoyi Guo, Liang Jin, Huifen Zhou, Yida Bao, Jiehong Yang, Jing Chen, Yu He, Daojun Yu, Haitong Wan

**Affiliations:** 1 Zhejiang Chinese Medical University, Hangzhou Zhejiang, 310053 PR China; 2 Affiliated Hangzhou First People’s Hospital, Zhejiang University of Medicine, Hangzhou, Zhejiang 310003 PR China

**Keywords:** *Multidrug-resistant Acinetobacter baumannii*, Glycyrrhetinic acid, Meropenem, Cell infection, Inflammation, Antioxidant

## Abstract

Glycyrrhetinic acid (GA) is a bio-effective component of Licorice. The GA is a monomer and the ingredient is an Oleanane-type pentacyclic triterpenes that has been used as a remedy for years. Due to the abuse of antibiotics, people pay attention to the emergence of *Multidrug-resistant Acinetobacter baumannii* (MDR-AB). As a conditional pathogen, MDR-AB causes severe infection, endangering human lives. Our previous studies found GA played an important role in Yinhua Pinggan, a Chinese medicine. However, whether GA could protect lung epithelium from MDR-AB-induced cell injury was elusive. Herein, we investigated the effects of GA on MDR-AB-infected A549 cells. The results showed GA had slightly antibacterial activity to MDR-AB in the GA (high concentration) but no impact on drug resistance genes. Notwithstanding, GA could reverse MDR-AB-induced cell apoptosis, hampered adhesion and invasion of MDR-AB to cells, and inhibit pro-inflammatory cytokines expression of IL-1β, IL-6, and TNF. Besides, MDR-AB-induced reactive oxygen species, pro-oxidative protein malonaldehyde, and myeloperoxidase of cells were decreased by GA, while antioxidative proteins were recovered, showing antioxidative capacity of GA might play a critical role. The expressions of toll-like receptor (TLRs) - 1, 2, 4, 5, 6, and 9 were increased by MDR-AB infection, while GA reversed the tendency. Interestingly, GA inhibited MDR-AB induced myeloiddifferentiationfactor88 expression (MYD88), one downstream con-factors of TLRs, but no affection on Interferon regulatory Factor 3 (IRF3), the other one, indicating GA inhibited MDR-AB induced cell injury by impact TLR/MYD88 pathway to attenuate inflammation. Altogether, our results demonstrated that GA protects against MDR-AB-induced cell injury through its antioxidative and anti-inflammatory properties, which deserve further study in the future.

**Supplementary Information:**

The online version contains supplementary material available at 10.1186/s40360-023-00648-z.

## Introduction

*Acinetobacter baumannii (AB)* was one of the most important hospital pathogens due to the abuse of antibiotics [1]. AB could be parasitic on the skin, respiratory tract, genitourinary system and mucous membrane of hospital patients and then infection [2, 3]. It could cause a broad spectrum of infections, such as bloodstream urethral infections, ventilator-associated pneumonia, wounded skin, and soft tissue infections, resulting in high morbidity and mortality for humans [4, 5]. Among them, ventilator-associated pneumonia and blood infections were the severest infection with the most increased mortality and were common susceptibility to patients with a poor medical condition or weakness [6–8].

Normally, the patients with AB infection were treated with antibiotics like imipenem, meropenem, and tigecycline in the clinic [9, 10]. However, owing to the irrational use of antibiotics in recent years, the drug resistance of *Multidrug-resistant Acinetobacter baumannii* (MDR-AB) has been more serious [11]. It had been reported that the mechanism of antibiotic resistance in MDR-AB involved β-lactamase production, efflux pump overexpression, biofilm formation and membrane pore permeability, protein binding site changes and other mechanisms [12–14]. Commonly, the resistant strain isolated from the clinic owned more than one antibiotic resistance mechanism, which resulted in multiply resistant to various antibiotics [15]. Therefore, finding new therapeutic approaches to confront MDR-AB infection is necessary and inevitable.

Thanks to its antibacterial, antiviral, anti-inflammatory, and sedative effects, Licorice has been a medicine for many years [16–19]. Glycyrrhetinic acid (GA) was the main effective component of Licorice [20]. GA has various pharmacological activities, including antibacterial, anti-inflammatory, antiviral, and other effects, and treating inflammatory diseases caused by microbial infection [21–23]. For instance, GA had anti-inflammatory effects on radiation-induced skin damage by inhibiting the activation of p38 mitogen activated protein kinase (p38MAPK) and nuclear factor kappa-B (NF-κB) signaling pathways, as well as the DNA-binding activity of P65 and activating protein-1 (AP-1), thereby inhibiting the production of pro-inflammatory cytokines [24]. GA could activate the Nuclear Factor erythroid 2-Related Factor 2 (Nrf2) antioxidant pathway in HepG2 cells and increase the expression of its target genes, which may be partly related to their protective role in TP-induced oxidative stress [25]. In addition, GA showed bactericidal activity against MRSA at sub-lethal doses by inhibiting virulence gene expression of *S. aureus* in vitro and in vivo [26]. GA was one of the putative additives of Traditional Chinese medicine (TCM) compounds, including Yinhua Pinggan Keli, Ephedra decoction, and Lianhua Qingwen, to treat respiratory infections such as influenza andpneumonia [27–29]. However, little was known about whether GA had antibacterial or protective effects during MDR-AB infection and induced cell injury.

Given all that, this study aimed to elucidate the effects of GA on MDR-AB infection in vitro. Initially, we firstly evaluated the antibacterial effects of GA against clinical MDR-AB strains directly. Then, we analyzed the protective effects of GA on MDR-AB-induced cell injury. Finally, the molecular mechanism of GA effects was investigated based on its anti-inflammatory and antioxidant properties.

## Materials and methods

### Reagents and materials

GA was provided by Chengdu Alpha Biotechnology Co., LTD with High-performance liquid chromatography ≥98%. The lung epithelial cell line A549 was purchased from the National Collection of Authenticated Cell Cultures. The reverse transcription kit and SYBR PreMix Ex Taq™II kit were supplied by Toyo Spinning (Shanghai, China). Tumor necrosis factor-α (TNF), Interleukin 6 (IL-6), interleukin 1-β (IL-1β) Enzyme-linked Immunosorbent Assay kit was purchased from Jiangsu Enzyme-labeled Biotechnology Co., LTD. The primer sequences were synthesized by Sangon Bioengineering (Shanghai) Co., LTD. Biyuntian Biotechnology Co., Ltd. provides reactive oxygen species (ROS) kit.

### The characteristics of MDR-AB

MDR-AB strain was isolated from the clinic. In order to detect the antibacterial activity of GA and antibiotics, we tested the Minimum Inhibitory Concentration (MIC) of antibiotics and GA. The MICs were determined by using the micro-dilution method. Briefly, MDR-AB was inoculated in LB liquid medium with or without indicated concentrations of Cefotaxime, Meropenem (MER), Cefalexin, Polymyxin B, and GA, then cultured at 37 °C for 24 h. After 24 h of culture, 100 ul of the bacterial solution was added to the 96-well plate, we measured the the optical density (OD) value to detect the bacterial concentration to determine the bacterial proliferation after the addition of the drug and OD value of samples was detected by a standard enzyme instrument (Molecular Devices, USA) at 600 nm.

### Cell culture

Due to high similarity and popular alternative for human primary pulmonary epithelial cells, A549 cells were selected in this study [30, 31]. Cells were seeded in a cell culture dish in F12K medium supplemented with 10% fetal bovine serum (FBS, Gibco, Poncho), with penicillin 100 μg/mL and streptomycin 100 μg/mL (Beyotime, Shanghai, China) and incubated at 37 °C in 5% CO_2_ and routinely digested with 0.25% pancreatic enzyme (containing ethylene diamine tetraacetic acid (EDTA) (Century, Hangzhou, China)). MOI was the ratio of the number of bacteria to the number of cells and could be used to indicate the number of bacteria added. The degree of cell damage can be detected by measuring the amount of lactate dehydrogenase (LDH) released, so the optimal multiplicity of infection (MOI) value was selected to establish a cell injury model with MDR-AB infection by LDH assay. In brief, the cell suspension was inoculated into the 24-well plate at a density of 1 × 10^5^ cells/mL infected with MDR-AB (MOI 15, 30, 60, 90, or 120) for 12 h. Afterward, the supernatant of each group was absorbed, centrifuged at 3000 RPM for 5 min, and 120 μL supernatant was placed in a 96-well plate. Reagents were added according to the instructions of LDH kit (Beyotime, Shanghai, China) and put at room temperature under dark conditions for 30 min, OD values at 490 nm were measured by standard enzyme instrument.

### Cytotoxicity of GA on A549 cells

Cells were inoculated into each well of the 96-well plate at 1 × 10^5^ cells/mL. Different concentrations of GA were added and cultured at 37 °C in 5% CO_2_ for 24 h. Afterward, the supernatant was replaced with containing 10% Cell Counting Kit-8 (CCK-8 (Beijing Zoman Biotechnology Co., Ltd., Beijing, China)) fresh cell culture medium in each well. After 2 h incubation at 37 °C, the OD value of stimulated luminescence was detected by enzyme standard instrument at 450 nm. Cell viability was determined as follow: Cell viability(%) = [A (experiment) -A (blank)]/ [A (control) -A (blank)] × 100%.

### The protective effects of GA on MDR-AB infected cells were assayed

A549 cells were cultured in an F12K medium and used as a model for MDR-AB infection. At least 1 × 10^5^ cells/mL A549 cells were cultured in 24-well culture plates before infection. The cells were divided into a control group, MDR-AB group (8 μg/mL), high concentration GA group (25 μmol/L), low concentration GA group (5 μmol/L). In addition to these groups, high concentration GA combined with MER group and low concentration GA combined with MER group were set up to study whether GA administration would affect the efficacy of MER. All groups, except for the control group, were incubated with or without MDR-AB (MOI 60) for 12 h, and cell injury rate was assayed with LDH experiments.

A549 cells were treated with the above-mention method and analyzed with the apoptotic kit (BD, USA) to investigate the effects of GA on MDR-AB-induced apoptosis. In brief, the supernatant was collected into a 15 mL centrifuge tube, the cells were washed with 1 mL PBS, and the washing liquid was added into the 15 mL centrifuge tube. The cells were digested and collected into a 15 mL centrifuge tube and centrifuged for 5 min. Discard the supernatant and re-suspend with PBS, centrifuged for 5 min. After washing with PBS, cells were re-suspended in 1X binding buffer. 2.5 μL of AnnexinV-FITC and 5 μL PI were added to 500 μL cell suspension and incubated for 15 min at room temperature in the dark. Finally, the samples were analyzed with a flow cytometer (CytoFLEX, USA).

To further evaluate the effects of GA on cell morphology with MDR-AB infection, we fixed the cells with 500 uL of 4% paraformaldehyde in a 6-well plate and were observed under an inverted microscope. The protective effect of GA on MDR-AB infected A549 cells was studied by observing the number and status of cells under inverted microscope.

### Bacterial adherence & invasion assay

A549 cells were treated as shown in 2.5, and after incubation for 12 h, non-adhered bacteria were removed by washing with PBS 3 times in the adhesion experiment. Cells were permeated by adding 250 μL 0.1% (V/V) Triton X-100, and cell lysates were collected. 100 μL 10-fold series diluents of cell lysates were prepared with PBS and incubated in LB agar plates for 24 h. Adhesion rate is expressed as the percentage of adherent bacteria relative to the number of all bacteria used in the experiment.

As to the invasion experiment, the cells were treated as above said, and after washing with PBS 3 times, 2 mL F12K medium containing polymyxin B was added to each well and incubated at 37 °C for 2 h to kill MDR-AB that did not invade into the cells. The invasion rate is expressed as the number of invasive bacteria relative to the total number of bacteria used in the experiment. We washed the cells with PBS three times, and the following steps were the same as the adhesion experiment.

### Enzyme-linked immunosorbent assay (ELISA)

The cell supernatant in each group was collected, and the contents of Interleukin-1β (IL-1β), Interleukin-6 (IL-6), and Tumor Necrosis Factor-α (TNF) in the cell supernatant of each group were detected by ELISA kit. The minimum concentration of IL-1β and TNF was 2.5 pg/mL. The minimum concentration of IL-6 was 1.5 pg/mL. The specific steps were strictly following the instructions of the ELISA kit.

### Quantitative reverse-transcription polymerase chain reaction (qRT-PCR)

Total RNA was extracted using the Trizol kit after grinding. The purity and concentration of RNA were detected using a micronuclei acid analyzer. RNA was reverse transcribed into cDNA using a Rever Tra Ace® qPCR RT Kit (Toyobo, Japan). The reaction system was prepared according to the instructions of the SYBR® Green Realtime PCR Master Mix (Toyobo, Japan). PCR amplification was performed, and each sample was repeated three times. Relative expression was calculated using the 2^-△△Ct^ method. Primer sequences are listed in Supplementary Table 1.

### Effects of GA on reactive oxygen species in infected MDR-AB

DCFH fluorescence assay measured the level of intracellular reactive oxygen species [32]. Cells were treated as shown in 2.5, then washed with PBS three times and added serum-free medium containing ROS probe for 20 min. Afterward, cells were washed with serum-free medium three times, and the results were performed with the flow cytometer. DCFH-DA can freely penetrate cell membranes.

### Statistical analysis

Statistical data analysis was performed using Prism 5.0 as means±standard error of the mean (SEM). The data represented three repeated experiments. The statistically significant differences were analyzed by the one-way ANOVA method. *P* < 0.05 is significant.

## Results

### Effects of GA on MDR-AB

**Fig. 1:**
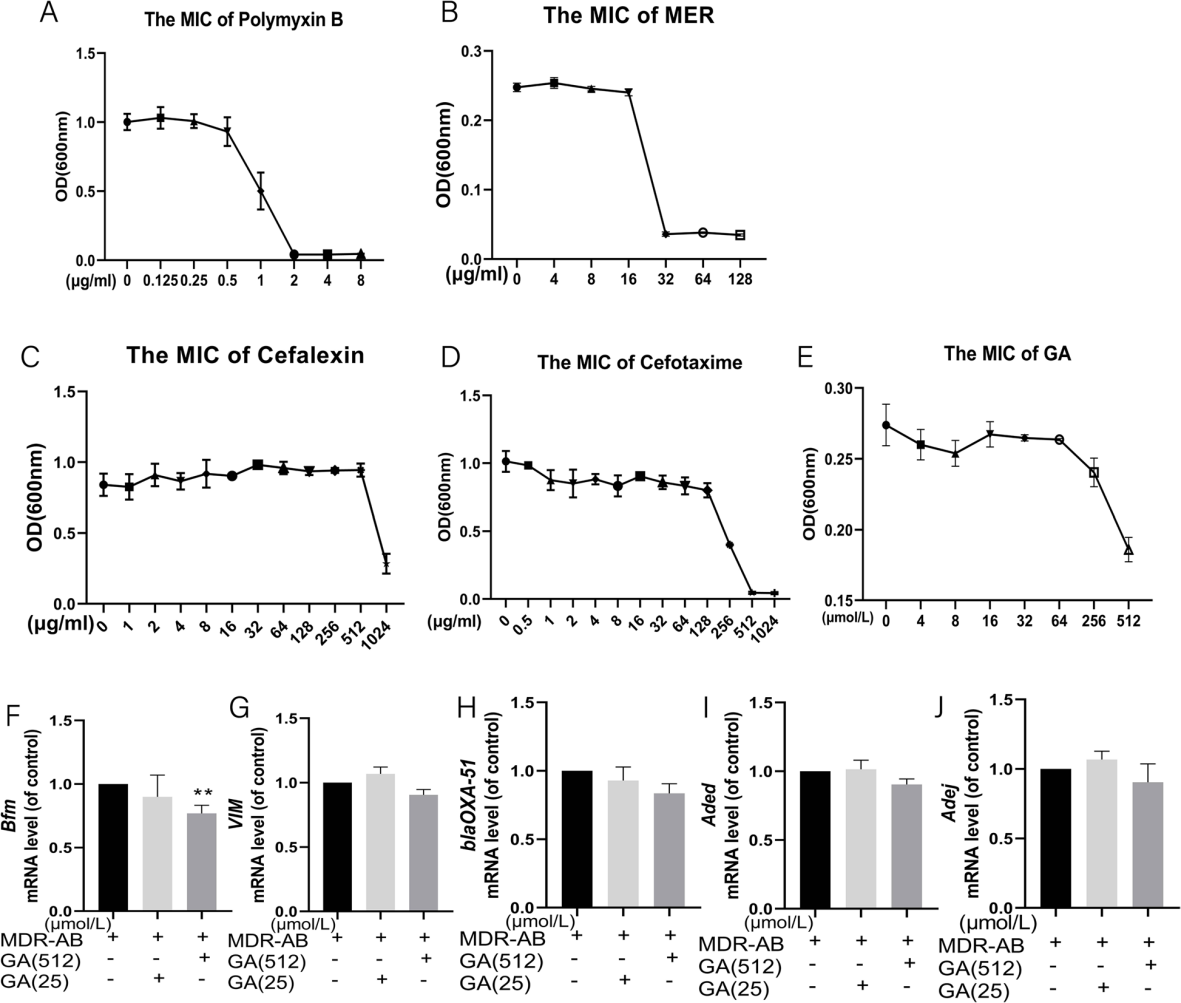
The study on drug resistance of MDR-AB strain. Polymyxins B (**A**), MER (**B**), Cefalexin (**C**), Cefotaxime (**D**), and different concentrations of GA (**E**) were incubated with MDR-AB for 24 h, respectively, and OD values were measured by microplate meter. The expression of *Bfm* (**F**), *VIM* (**G**), *blaOXA-51* (**H**), *Aded* (**I**), and *Adej* (**J**) were detected by RT-PCR after GA and MDR-AB were co-cultured for 24 h.^*^P < 0.05 vs. control group; ^**^P < 0.01 vs. control group

Firstly, the selected MDR-AB strain was analyzed by drug sensitivity test. As shown in Fig. 1A-D, MIC values of cefotaxime and cefalexin were both greater than 512 μg/mL, MIC of MER were greater than 32 μg/mL, and MIC of polymyxins B were both less than 2 μg/mL, indicating that the MDR-AB screened by us was carbapenems resistant bacteria following the standards of the Clinical and Laboratory Standards Institute (CLSI) 2021-M100. As the maximum dissolved concentration of GA in LB medium was at 1024 μmol/L, it had only a certain antibacterial effect (Fig. 1E). To elucidate the impact of GA resistance genes, we further studied the expression of related genes by RT-PCR. The results showed that the expressions of *VIM*, *blaOXA-5*, *Aded*, and *Adej* were not significantly down-regulated at 25 μmol/L and 512 μmol/L of GA compared with the control group, but the expression of *Bfm* was significantly down-regulated at 512 μmol/L of GA (Fig. 1F-J). Together, these results showed GA has a partial bacteriostatic effect on MDR-AB, but the direct impact of GA on MDR-AB was not observed on other resistant genes, except *Bfm*, in the GA (high concentration).

### Effects of GA on cytotoxicity of A549 cells

**Fig. 2:**
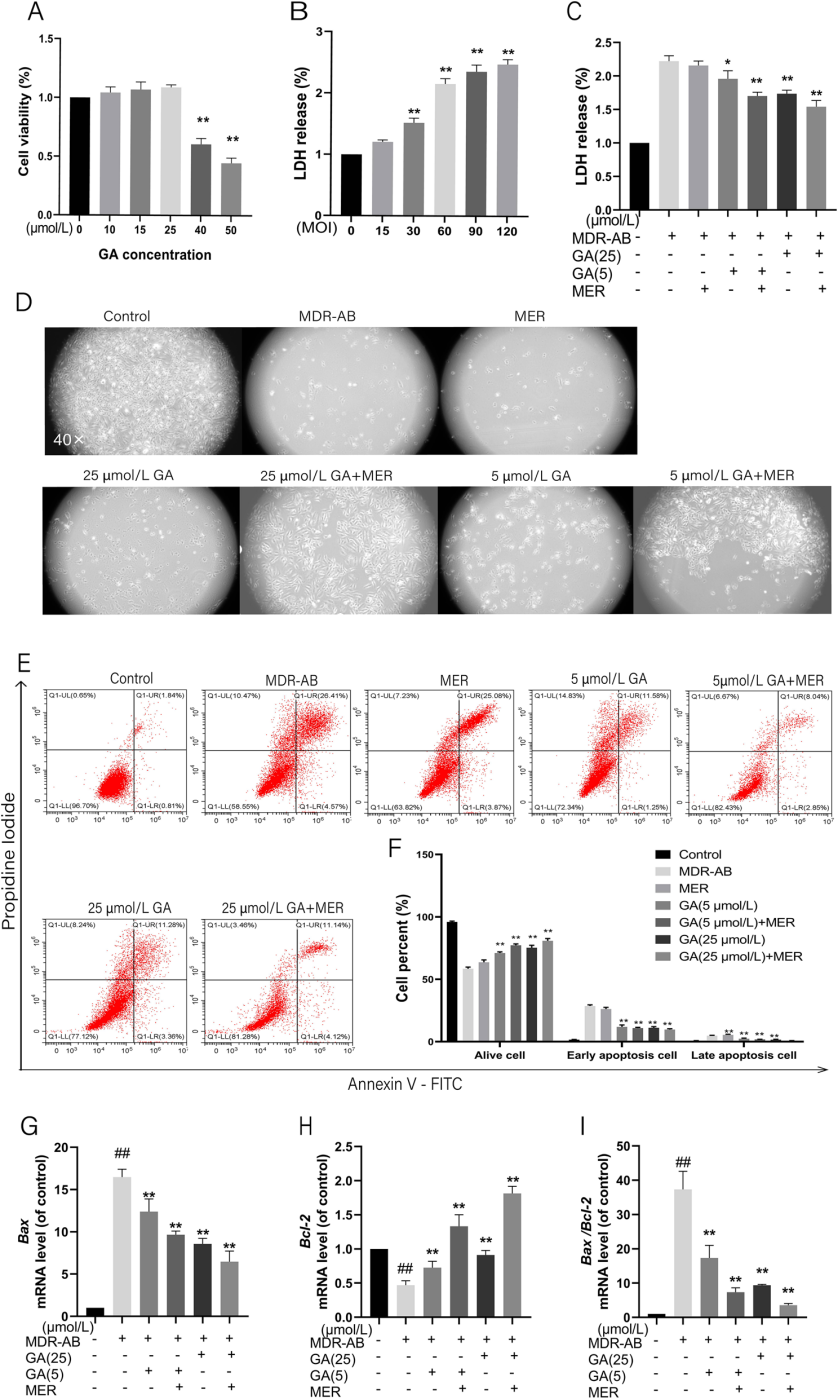
The effects of GA on MDR-AB infected cell injury. Different concentrations of GA were added into A549 cells, and cytotoxicity was detected by CCK8 assay (**A**). Various concentrations of MDR-AB were added to A549 cells, and cytotoxicity was measured by LDH (**B**). The effect of GA on the cell viability of MDR-AB-infected A549 cells was measured by LDH (**C**). Cell morphology was observed after MDR-AB invasion for 12 h by inverted microscope (**D**). Apoptosis was detected by flow cytometry (**E**, **F**), and the mRNA expression level of *Bcl-2* and *Bax* was detected by RT-PCR (**G**-**I**). ^*^P < 0.05 vs. MDR-AB group; ^**^P < 0.01 vs. MDR-AB group; ^#^*P* < 0.05 vs. control group; ^##^*P* < 0.01 vs. control group

A549 cells were treated with different concentrations of GA for 24 h, and the survival rate of A549 cells was detected by CCK-8 assay. There was no significant difference in cell survival rate in groups of 10 μmol/L, 15 μmol/L, and 25 μmol/L, but it significantly decreased in groups of 40 μmol/ L and 50 μmol/L (Fig. 2A) compared with the control group. Therefore, 25 μmol/L of GA was the maximum innocuous concentration for drug administration. Thus, we used 5 μmol/L of GA as the low concentration and 25 μmol/L of GA as the high concentration in the following study.

### Effects of GA on cell viability of MDR-AB induced A549 cell injury

The best MOI of MDR-AB to induce inflammatory response was firstly selected. The MDR-AB of MOI 15, 30, 60, 90, and 120 were used to stimulate A549 cells for 12 h, respectively. LDH results showed that the cell survival rate in the MOI 30, 60, 90 and 120 group was significantly reduced compared with that of the control group (Fig. 2B). In the literature, the bacterial concentration with a fatality rate of 50% was generally selected as the experimental concentration [33, 34].. Therefore, we selected MOI 60 for subsequent experiments. Then, the GA (high concentration), GA (low concentration), and GA + MER were used to interfere with MDR-AB-infected A549 cells for 12 h. As shown in Fig. 2C, the cell viability of A549 cells in group MDR-AB was significantly decreased, but the GA with or without MER reversed it, particularly in the GA (high concentration) compared with the control group. Besides, the cell morphology was observed under an inverted microscope. As shown in Fig. 2D, the number of living cells was decreased, and apoptosis was observed during MDR-AB infection. The apoptotic cells were significantly reduced after adding GA. Although there was no difference between MER and MDR-AB, the combination of MER and GA also reversed MDR-AB-induced apoptosis (Fig. 2D). These results indicated that GA and MER had a protective effect on MDR-AB-induced cell injury.

The apoptotic rate of A549 cells in the MDR-AB group was significantly higher than that of the control group. But the cell viability in the GA group and GA + MER group increased considerably, particularly in the GA (high concentration) + MER group (Fig. 2E and F) compared with the MDR-AB group. To further assay the apoptosis, Annexin V/PI was used to determine the effect of GA on MDR-AB-induced apoptosis. In addition, RT-PCR was performed to detect the mRNA expression levels of apoptotic protein *Bax* and anti-apoptotic protein *Bcl-2*, and as shown in Fig. 2G-I, the expression of Bax in the MDR-AB group was increased, while the expression of *Bcl-2* was decreased compared with the control group. However, *Bax* expression in both the GA and GA+ MER groups decreased, while *Bcl-2* was increased compared with the MDR-AB group. These results suggested GA had a protective effect on MDR-AB-involved apoptosis and synergetic function with MER.

### Effects of GA on adhesion and invasion of MDR-AB

**Fig. 3:**
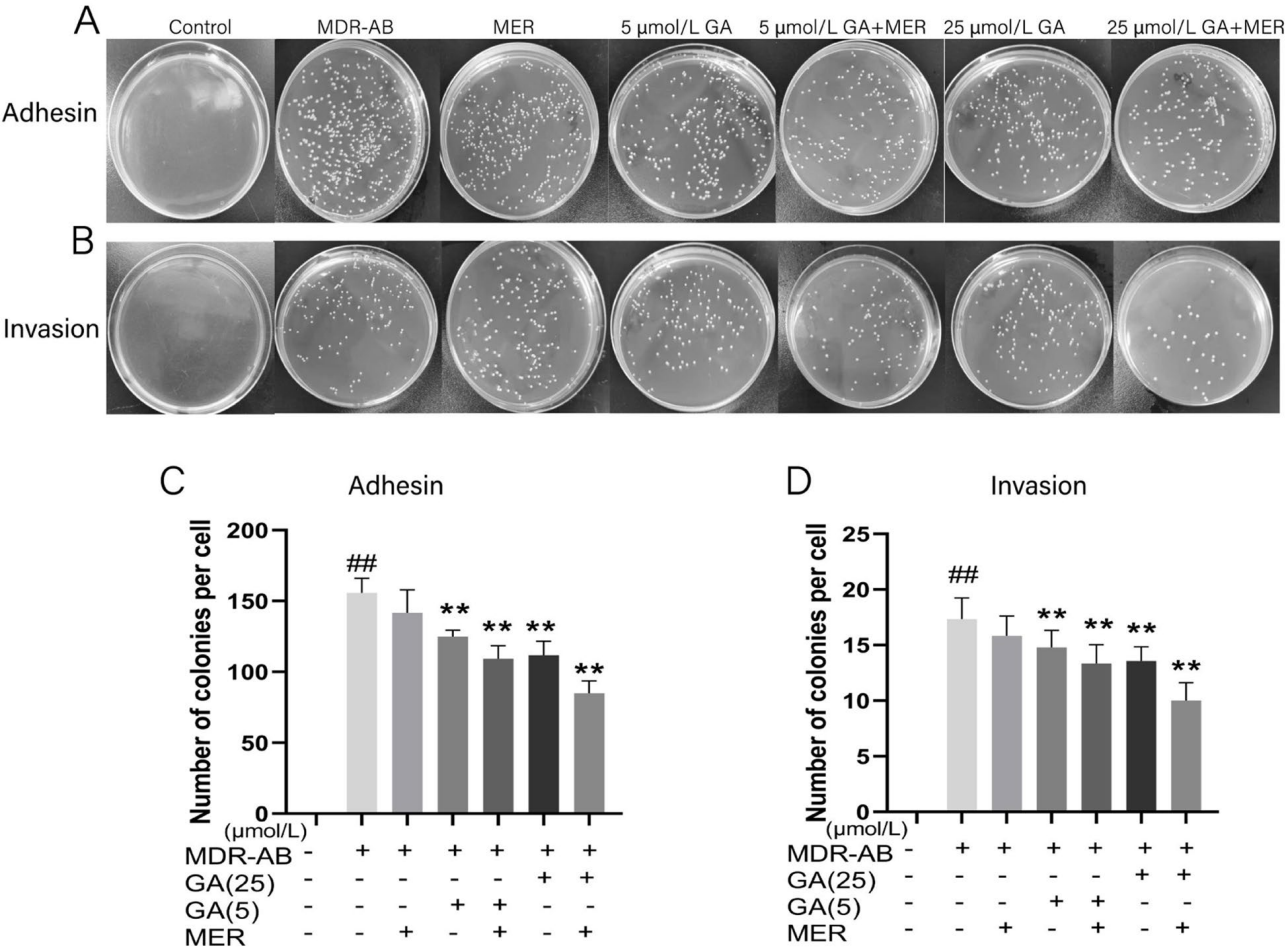
Effects of GA on adhesion and invasion of MDR-AB infecting cells. MDR-AB infecting Cell was cultured with or without GA and/or MER for 12 h, and the supernatant of each group of cells was discarded and washed with PBS three times. The culture medium was discarded, and the cells were lysed. The lysate was diluted and incubated on LB solid medium for counting adhered (**A**, **C**) and invaded bacterially (**B**, **D**). ^*^P < 0.05 vs*.* MDR-AB group*;* ^*^P < 0.05 vs. MDR-AB group; ^**^P < 0.01 vs. MDR-AB group: ^#^*P* < 0.05 vs. control group; ^##^*P* < 0.01 vs. control group

Next, we analyzed the effects of GA or MER combined with GA on the adhesion and invasion of MDR-AB by plate counting. The numbers of adhered (Fig. 3A and C) or invaded (Fig. 3B and D) bacteria in the MER group have no obvious differences, but the various concentrations of the GA and MER groups were significantly reduced, respectively, particular in the GA + MER group compared with group MDR-AB. Our result demonstrated that GA could dramatically inhibit the invasion and adhesion of MDR-AB to A549 cells, and it also had a synergistic effect with MER.

### Effects of GA on MDR-AB induced inflammatory cytokine secretion

**Fig. 4:**
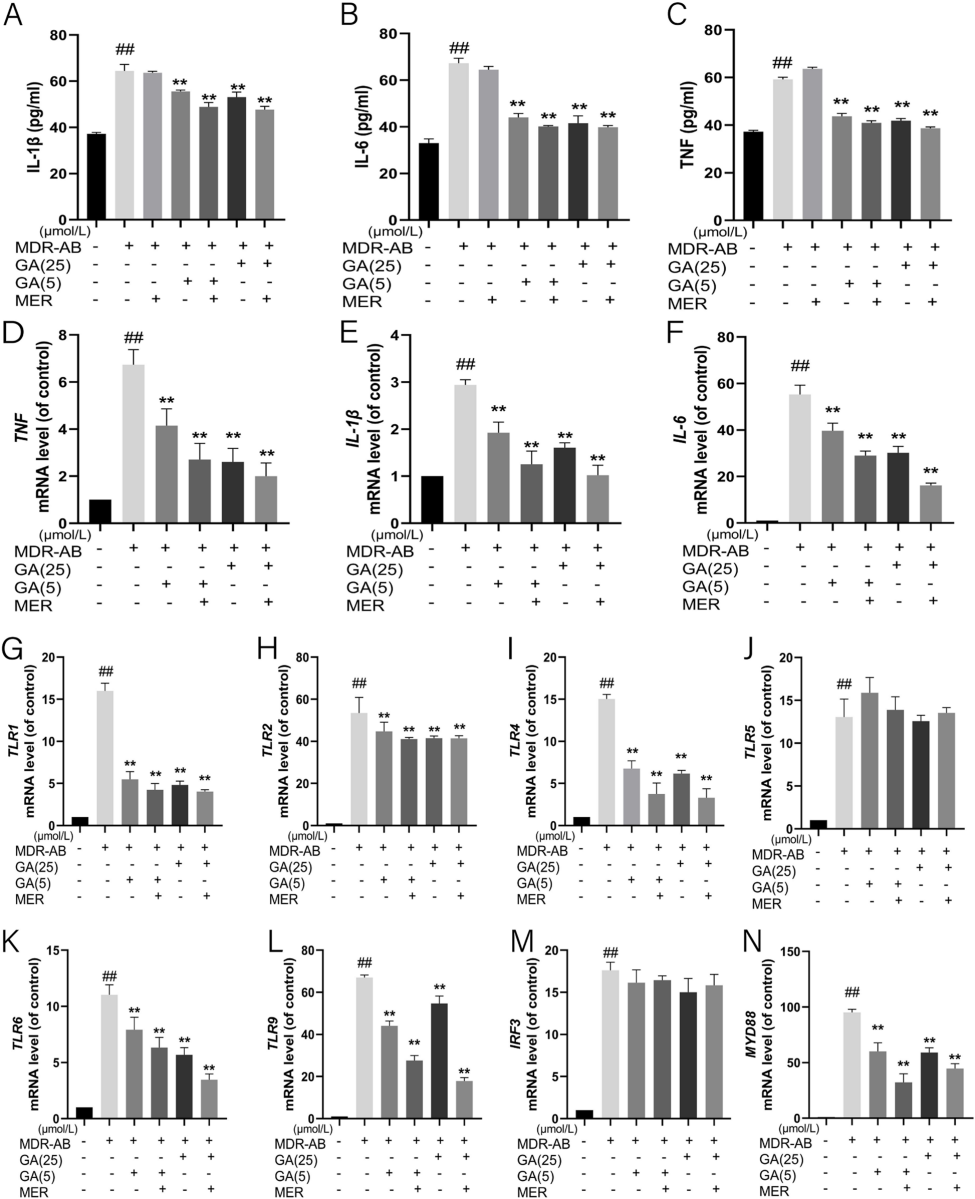
Effect of GA on MDR-AB infected inflammation of A549 cells. The contents of IL-1β (**A**), IL-6 (**B**), and TNF (**C**) in the supernatant was determined by ELISA after 12 h culture. After 12 h of culture, the supernatant was discarded, and cells were collected. The mRNA expression levels of *TNF* (**D**), *IL-1β* (**E**), *IL-6* (**F**), *TLR-1* (**G**), *TLR-2* (**H**), *TLR-4* (**I**), *TLR-5* (**J**), *TLR-6* (**K**), *TLR-9* (**L**), *IRF3* (**M**) and *MYD88* (**N**) were detected by RT-PCR. ^*^P < 0.05 vs. MDR-AB group; ^**^P < 0.01 vs. MDR-AB group: ^#^*P* < 0.05 vs. control group; ^##^*P* < 0.01 vs. control group

To analyze the effects of GA on MDR-AB-induced pro-inflammatory cytokines. As shown in Fig. 4A-C, the contents of TNF, IL-1β, and IL-6 in the group of MDR-AB were significantly increased compared with that of the control group, while these cytokines in the GA (high concentration), GA (low concentration) and GA + MER group were significantly decreased compared with that of MDR-AB the group. To further investigate the effects of GA on MDR-AB-induced inflammation, we used an RT-PCR assay to detect the mRNA expression level of these proteins, and the results are similar to the ELISA assay (Fig. 4D-F).

Given the anti-inflammatory effect of GA, we further explored its signaling pathway. TLRs receptors were one of the most important pro-inflammatory pathways [35]. Thus, the TLRs related genes such as *TLR-1*, *TLR-2*, *TLR-4*, *TLR-5*, *TLR-6,* and *TLR-9* were detected by RT-PCR assay in this study. As shown in Fig. 4G-L, the mRNA expression of inflammatory factors in group MDR-AB was significantly increased compared with the control group*.* After GA treatment, the expression levels of *TLR-1*, *TLR-2*, *TLR-4*, *TLR-6,* and *TLR-9* were decreased considerably, but the expression levels of *TLR-5* show no difference compared with the MDR-AB group. Then, we detected the mRNA levels of *MYD88* and *IRF3* and found that *MYD88* expression decreased significantly after GA treatment, while *IRF3* did not decrease significantly compared with the MDR-AB group (Fig. 4M and N). These results noted that GA inhibited inflammation by inhibiting the expression of TLR/MYD88/NF-κB, thereby reducing cell apoptosis.

### Effects of GA on MDR-AB-induced ROS

**Fig. 5:**
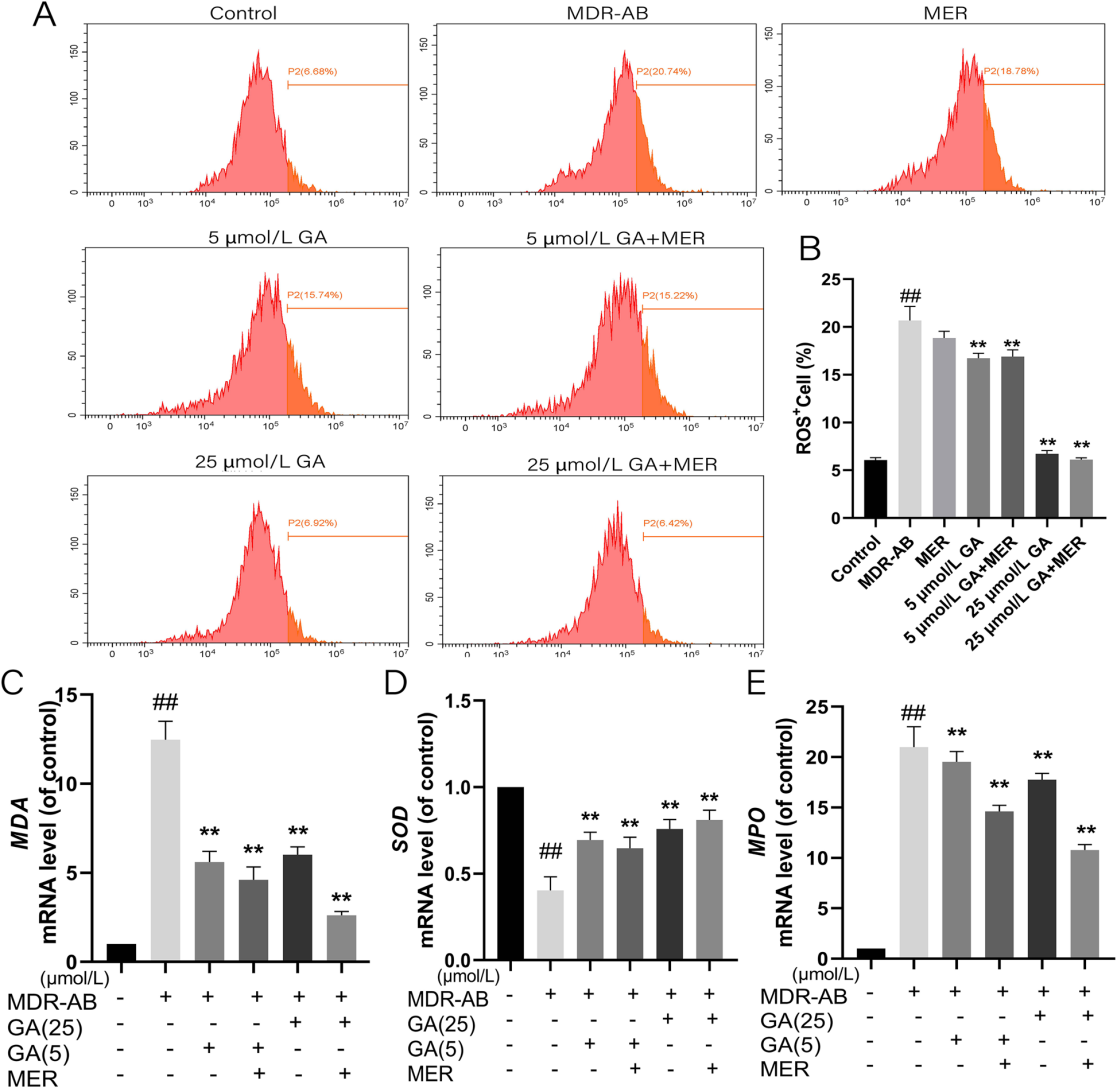
Effects of GA on MDR-AB induced ROS expression of A549 cell. After 12 h of culture, the cells were labeled with DCFH-DA, and the ROS^+^ cell percentages were detected by flow cytometry (**A**, **B**). The mRNA expression of *MDA* (**C**), *SOD* (**D**), and *MPO* (**E**) were detected by RT-PCR assay. ^*^P < 0.05 vs. MDR-AB group; ^**^P < 0.01 vs. MDR-AB group: ^#^*P* < 0.05 vs. control group; ^##^*P* < 0.01 vs. control group

We measured ROS concentration using flow cytometry to investigate whether GA can affect ROS expression induced by MDR-AB infection. The ROS expression in the MDR-AB group was significantly increased compared with the control group. The content of ROS in the groups of the GA (high concentration) and GA (low concentration), and GA + MER were significantly decreased (Fig. 5A and B) compared with the group of MDR-AB. We detected the expression of antioxidant-related proteins through PCR experiments and found that after MDR-AB infected A549 cells, the expression levels of *MDA* and *MPO* increased, while the expression levels of SOD decreased. After GA treatment, the expression levels of *MDA* and *MPO* decreased, and the expression level of *SOD* increased (Fig. 5C-E) compared with the MDR-AB group. In conclusion, GA can regulate ROS levels, reduce the expression level of *MDA* and *MPO*, and improve the expression level of *SOD*, which might play an antioxidant role during the MDR-AB infection.

## Discussion

This study found that GA had a partial bacteriostatic effect on MDR-AB directly, but RT-PCR showed that GA had no significant impact on MDR-AB drug resistance genes. Furthermore, we reported the protective effect of GA on MDR-AB-induced cell damage. The apoptosis and oxidative damage induced by MDR-AB were reduced in the presence of GA. We found that GA could inhibit the adhesion and invasion ability of MDR-AB to cells. In addition, Its anti-inflammatory effects were confirmed by inhibiting TLRs/MYD88 signaling pathway while its antioxidative effects were elucidated by reducing MDR-AB induced ROS expression.

Previous data indicated that the ability of bacteria to invade cells was an effective external expression of virulence [36]. Some studies suggested GA could inhibit the growth of *Staphylococcus aureus* in vitro in a dose-dependent bactericidal effect [37, 38]. Moreover, GA could hinder the formation of MDR-AB biofilm and affect the overall structure of biofilm [39]. In this study, we found GA could inhibit the proliferation of MDR-AB but had no effect on drug resistance genes.

After bacterial infection, the host could respond to pathogen infection in various ways, such as inducing inflammatory response and activating apoptosis-related signaling pathways [40]. Studies have shown that AB infection led to changes in the growth state of lung epithelial cells and enhanced programmed cell death [41]. Polyvinylpyrrolidone-capped silver nanoparticles could play a protective role by reducing AB-induced cell death and decreasing the intracellular activity of AB [42]. In addition, GA could reduce cell death and play a protective role in the co-culture of staphylococcus aureus and A549 cells [38]. In this study, we found that as long as MDR-AB infected A549 cells, cell viability would be decreased, cell morphology would change, and the number of apoptosis increase. However, after GA treatment, cell viability was significantly increased, and the number of apoptosis was decreased. These results suggested that GA had a protective effect on MDR-AB-infected A549 cells. At the same time, we confirmed that GA can also reduce the adhesion and invasion ability of MDR-AB, and showed a protective effect.

Previous data proved that *Rifampicin* could achieve protective effects by reducing AB apoptosis, oxidative stress, and pro-inflammatory cytokine release [33]. As to bacteria-induced inflammation, GA could exert its anti-inflammatory activity by down-regulating NO, pro-inflammatory cytokines, chemokines, and anti-inflammatory cytokines, and the anti-inflammatory mechanism may involve inhibition of NF-κB, mitogen-activated protein kinase (MAPKs) and phosphatidylinositol-3-kinases (PI3K) /protein kinase B (PKB) related inflammatory signaling pathways and Nrf2 / heme oxygenase 1 (HO-1) signaling pathway activation [21]. In this study, we found that MDR-AB could activate the NF-κB signaling pathway by binding to TLRs receptors, and increased the expression of inflammatory cytokines IL-1β, IL-6 and TNF. But GA could inhibit NF-κB and MYD88 expression and decrease the expression of pro-inflammatory proteins. Excessive accumulation of intracellular ROS would also damage cell components, thereby happening apoptosis and other cell death processes. In our study, we found the expression mRNA level of *SOD* was decreased, and both *MDA* and *MPO* were increased amid MDR-AB infected A549 cells, while GA treatment could increase the expression mRNA level of *SOD* and decrease both *MDA* and *MPO*. Therefore, GA might play an antioxidant role and increase the expression of antioxidant proteins.

However, this study still had some limitations. Firstly, although GA could inhibit oxidative stress induced by MDR-AB, the mechanism of oxidation resistance in this study had not been further studied. Secondly, this study only conducted experiments on cell lines and needed to confirm these results with primary cells. Finally, this study was only carried out *in vitro*, and animal experiments are required to be performed in the future.

## Conclusion

**Fig. 6:**
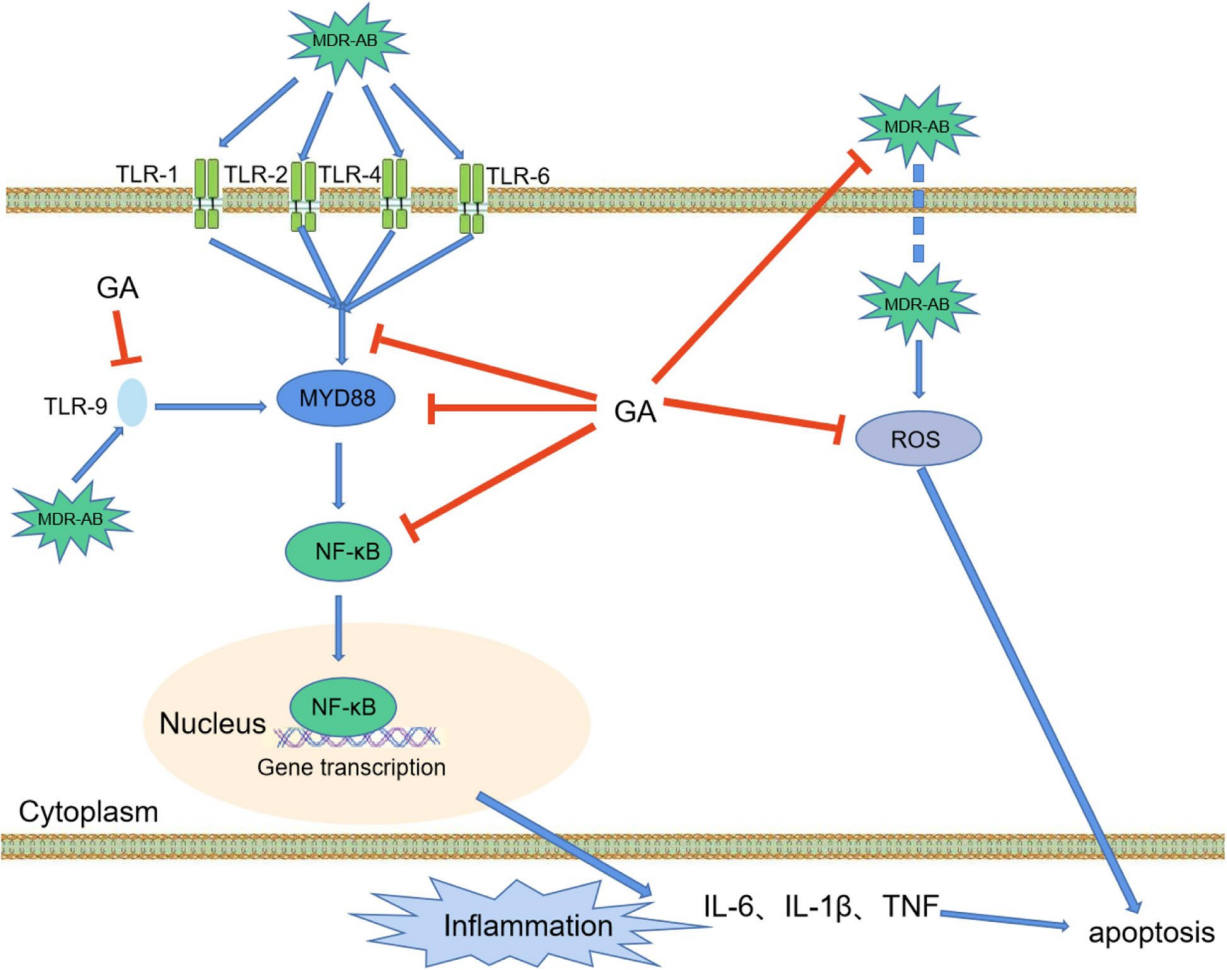
The mechanic diagram of GA to protect against MDR-AB-induced cell injury. On the one hand, MDR-AB activates MYD88 by binding TLR-1, 2,4,6, and intracellular TLR-9 receptors. MYD88 activates NF-κB, and the latter trans-locates into the nucleus, resulting in the expression of inflammatory factors. On the other hand, MDR-AB can invade cells, resulting in intracellular ROS expression. Thus, excessive inflammatory cytokines and ROS accumulation eventually lead to apoptosis, while GA can reverse the trend by inhibiting inflammation and oxidative stress

In this study, we reported that GA had a protective effect on A549 cells from MDR-AB invasion at an appropriate concentration. The GA protective effect was found to maybe through inhibition of the TLR/MYD88/NF-κB inflammatory pathway and reducing intracellular ROS expression, thereby reducing cell injury (Fig. 6). The results showed that GA could effectively reduce cell damage caused by MDR-AB infection of A549 cells. These results expand the knowledge of the molecular events underlying the protective action of GA in the context of pneumonia and provide new ideas and methods for the subsequent clinical treatment of diseases due to MDR-AB infection.

## Supplementary Information

**Additional file 1: Supplementary Table 1.** List of primers

## Data Availability

The datasets used and/or analyzed during the current study are available from the corresponding author on reasonable request.

## References

[1] Harding CM, Hennon SW, Feldman MF (2018). Uncovering the mechanisms of Acinetobacter baumannii virulence. Nat Rev Microbiol.

[2] Trebosc V, Gartenmann S, Tötzl M, et al. Dissecting Colistin Resistance Mechanisms in Extensively Drug-Resistant Acinetobacter baumannii Clinical Isolates. mBio. 2019; 10 2019/07/18. 10.1128/mBio.01083-19. 10.1128/mBio.01083-19 PMC6635527 31311879

[3] Li FJ, Starrs L, Burgio G. Tug of war between Acinetobacter baumannii and host immune responses. Pathogens Dis. 2018;76 2019/01/19. 10.1093/femspd/ftz004. 10.1093/femspd/ftz004 30657912

[4] Longo F, Vuotto C, Donelli G (2014). Biofilm formation in Acinetobacter baumannii. New Microbiol.

[5] Lee CR, Lee JH, Park M (2017). Biology of acinetobacter baumannii: pathogenesis, antibiotic resistance mechanisms, and prospective treatment options. Front Cell Infect Microbiol.

[6] Antunes LC, Visca P, Towner KJ (2014). Acinetobacter baumannii: evolution of a global pathogen. Pathogens Dis.

[7] Jean SS, Chang YC, Lin WC, et al. Epidemiology, Treatment, and Prevention of Nosocomial Bacterial Pneumonia. J Clin Med. 2020;9 2020/01/23. 10.3390/jcm9010275. 10.3390/jcm9010275 PMC7019939 31963877

[8] Michalopoulos A, Falagas ME (2010). Treatment of Acinetobacter infections. Expert Opin Pharmacother.

[9] Evans BA, Hamouda A, Amyes SG (2013). The rise of carbapenem-resistant Acinetobacter baumannii. Curr Pharmaceut Design.

[10] Ibrahim S, Al-Saryi N, Al-Kadmy IMS (2021). Multidrug-resistant Acinetobacter baumannii as an emerging concern in hospitals. Mole Biol Rep.

[11] Ramirez MS, Bonomo RA, Tolmasky ME. Carbapenemases: Transforming Acinetobacter baumannii into a Yet More Dangerous Menace. Biomolecules. 2020;10 2020/05/10. 10.3390/biom10050720. 10.3390/biom10050720 PMC7277208 32384624

[12] Say Coskun US, Caliskan E, Copur Cicek A (2019). β-lactamase genes in carbapenem resistance Acinetobacter baumannii isolates from a Turkish university hospital. J Infect Dev Countries.

[13] Opazo AC, Mella SM, Domínguez MY (2009). Multi-drug efflux pumps and antibiotic resistance in Acinetobacter baumannii. Rev Chilena Infectol.

[14] Mea HJ, Yong PVC, Wong EH (2021). An overview of Acinetobacter baumannii pathogenesis: Motility, adherence and biofilm formation. Microbiol Res.

[15] Zavascki AP, Carvalhaes CG, Picão RC (2010). Multidrug-resistant Pseudomonas aeruginosa and Acinetobacter baumannii: resistance mechanisms and implications for therapy. Expert Rev Anti Infect Ther.

[16] Xiang M, Zhou X, Luo TR (2019). Design, Synthesis, Antibacterial Evaluation, and Induced Apoptotic Behaviors of Epimeric and Chiral 18β-Glycyrrhetinic Acid Ester Derivatives with an Isopropanolamine Bridge against Phytopathogens. J Agricult Food Chem.

[17] Oyama K, Kawada-Matsuo M, Oogai Y (2016). Antibacterial Effects of Glycyrrhetinic Acid and Its Derivatives on Staphylococcus aureus. PloS one.

[18] Yang R, Wang LQ, Yuan BC (2015). The Pharmacological Activities of Licorice. Planta Medica.

[19] Lu Q, Wu X, Han W (2021). Effect of Glycyrrhiza uralensis against ulcerative colitis through regulating the signaling pathway of FXR/P-gp. Am J Transl Res.

[20] Yang R, Yuan BC, Ma YS (2017). The anti-inflammatory activity of licorice, a widely used Chinese herb. Pharmaceut Biol.

[21] Tu B, Liang J, Ou Y (2022). Novel 18β-glycyrrhetinic acid derivatives as a Two-in-One agent with potent antimicrobial and anti-inflammatory activity. Bioorganic chemistry.

[22] Baltina LA, Lai HC, Liu YC (2021). Glycyrrhetinic acid derivatives as Zika virus inhibitors: Synthesis and antiviral activity in vitro. Bioorgan Med Chem.

[23] Bian M, Zhen D, Shen QK (2021). Structurally modified glycyrrhetinic acid derivatives as anti-inflammatory agents. Bioorgan Chem.

[24] Su L, Wang Z, Huang F (2018). 18β-Glycyrrhetinic acid mitigates radiation-induced skin damage via NADPH oxidase/ROS/p38MAPK and NF-κB pathways. Environ Toxicol Pharmacol.

[25] Cao LJ, Li HD, Yan M (2016). The Protective Effects of Isoliquiritigenin and Glycyrrhetinic Acid against Triptolide-Induced Oxidative Stress in HepG2 Cells Involve Nrf2 Activation. Evid Based Complement Altern Med.

[26] Weaver AJ Jr, Borgogna TR, O'Shea-Stone G, et al. 18β-Glycyrrhetinic Acid Induces Metabolic Changes and Reduces Staphylococcus aureus Bacterial Cell-to-Cell Interactions. Antibiotics (Basel, Switzerland). 2022:11. 2022/06/25. 10.3390/antibiotics11060781. 10.3390/antibiotics11060781 PMC9220049 35740189

[27] Wu L, Chen Y, Ma Y (2020). Clinical practice guideline on treating influenza in adult patients with Chinese patent medicines. Pharm Res.

[28] Du HX, Zhou HF, He Y (2018). Immunologic mechanisms of Yinhua Pinggan granule and San-ao decoction against influenza virus in vivo. Zhongguo Zhong Yao Za Zhi.

[29] Ma YM, Zhao LJ, Liu MR (2021). Multiple components of Mahuang Shengma Decoction on prevention and treatment of acute lung injury based on RAGE/NF-κB signaling pathway. Zhongguo Zhong Yao Za Zhi.

[30] Foster KA, Oster CG, Mayer MM (1998). Characterization of the A549 cell line as a type II pulmonary epithelial cell model for drug metabolism. Experiment Cell Res.

[31] Jia XB, Zhang Q, Xu L (2021). Lotus leaf flavonoids induce apoptosis of human lung cancer A549 cells through the ROS/p38 MAPK pathway. Biol Res.

[32] Eruslanov E, Kusmartsev S (2010). Identification of ROS using oxidized DCFDA and flow-cytometry. Methods Mole Biol (Clifton, NJ).

[33] Smani Y, Domínguez-Herrera J, Pachón J (2011). Rifampin protects human lung epithelial cells against cytotoxicity induced by clinical multi and pandrug-resistant Acinetobacter baumannii. J Infect Dis.

[34] Wen SH, Lin LN, Wu HJ (2018). TNF-α increases Staphylococcus aureus-induced death of human alveolar epithelial cell line A549 associated with RIP3-mediated necroptosis. Life Sci.

[35] Kawai T, Akira S (2007). TLR signaling. Sem Immunol.

[36] Pizarro-Cerdá J, Cossart P (2006). Bacterial adhesion and entry into host cells. Cell.

[37] Long DR, Mead J, Hendricks JM (2013). 18β-Glycyrrhetinic acid inhibits methicillin-resistant Staphylococcus aureus survival and attenuates virulence gene expression. Antimicrobial Agents Chemother.

[38] Li HE, Qiu JZ, Yang ZQ (2012). Glycyrrhetinic acid protects mice from Staphylococcus aureus pneumonia. Fitoterapia.

[39] Paul Bhattacharya S, Mitra A, Bhattacharya A (2020). Quorum quenching activity of pentacyclic triterpenoids leads to inhibition of biofilm formation by Acinetobacter baumannii. Biofouling.

[40] Alamdary SZ, Bakhshi B, Soudi S (2018). The anti-apoptotic and anti-inflammatory effect of Lactobacillus acidophilus on Shigella sonnei and Vibrio cholerae interaction with intestinal epithelial cells: A comparison between invasive and non-invasive bacteria. PloS One.

[41] Smani Y, Docobo-Pérez F, McConnell MJ (2011). Acinetobacter baumannii-induced lung cell death: role of inflammation, oxidative stress and cytosolic calcium. Microbial Pathogenesis.

[42] Tiwari V, Tiwari M, Solanki V (2017). Polyvinylpyrrolidone-Capped Silver Nanoparticle Inhibits Infection of Carbapenem-Resistant Strain of Acinetobacter baumannii in the Human Pulmonary Epithelial Cell. Front Immunol.

