# Supplementary material for: Glycyrrhetinic acid protects against Multidrug-resistant Acinetobacter baumannii-induced lung epithelial cells injury by regulating inflammation and oxidative stress
Source: BMC Pharmacol Toxicol. 2023 Jan 30;24:5. doi: 10.1186/s40360-023-00648-z (PMC9887834; doi:10.1186/s40360-023-00648-z)
Supplement: Supplementary file 1 — Additional file 1: Supplementary Table 1. List of primers [file 40360_2023_648_MOESM1_ESM.docx]

**Supplementary Table 1 List of primers**

| Gene | Forward (5′-3′) | Reverse (5′-3′) |
| --- | --- | --- |
| **A549 cell** |  |  |
| *MYD88* | GCCGCCTGTCTCTGTTCTTGAAC | GGTCCGCTTGTGTCTCCAGTTG |
| *IRF3* | GAGGCTCGTGATGGTCAAGGTTG | AGTGGGTGGCTGTTGGAAATGTG |
| *Bax* | ACAGGGGCCCTTTTGCTTC | ACTCGCTCAGCTTCTTGGTG |
| *Bcl-2* | TCGCCCTGTGGATGACTGAGTAC | ACAGCCAGGAGAAATCAAACAGAGG |
| *SOD* | GATGACTTGGGCAAAGGTGGAAATG | CCAATTACACCACAAGCCAAACGAC |
| *GAPDH* | ACGGATTTGGTCGTATTGGG | TGATTTTGGAGGGATCTCGC |
| *TLR1* | CTCTGCTGATCGTCACCATCGTTG | TCCACTGGCACACCATCCTGAG |
| *TLR2* | CTACCAGATGCCTCCCTCTTACCC | ACCAGCTTCCAAAGTCTTCAGTGTG |
| *TLR4* | GCTCTTGGTGGAAGTTGAACGAATG | CAAGCACACTGAGGACCGACAC |
| *TLR5* | ATGTGGCTTGACGTTTCGGGGG | GCCGTGGATAATGGTTTACAATTCGGC |
| *TLR6* | TTGTCCCTGGCAAGAGCATTGTG | GTTCGTAATGGCACCACTCACTCTG |
| *TLR9* | GCATCTCGCAGGCAGTCAATGG | CCGTGAATGAGTGCTCGTGGTAG |
| *TNF* | GTGATCGGCCCCCAGAGGGA | CACGCCATTGGCCAGGAGGG |
| *IL-6* | CCACTCACCTCTTCAGAAC | CTTTGCTGCTTTCACACAT |
| *IL-1β* | GGACAGGATATGGAGCAACAAGTGG | TCATCTTTCAACACGCAGGACAGG |
| *MDA* | CCGCTATCTCATCTCGTGCTTCAG | AGGCAGAAAGGTCAGGTAGTCCAG |
| **MDR-AB** |  |  |
| *bla OXA-51* | TAATGCTTTGATCGGCCTTG | TGGATTGCACTTCATCTTGG |
| *VIM* | ATTGGTCTATTTGACCGCGTC | TGCTACTCAACGACTGAGCG |
| *Adej* | CTTGGTGTAACTGCCGGATT | TGAGCACCAGACTCACGTTC |
| *Aded* | TTGGCTTGCCAAATGACGTG | TCTTGACGACTAACCGCACC |
| *Bfm* | ATATATGCGGGGCTGGTAATTC | ATGCAGGTGCTTTTTTATTGGT |
| *GltA* | AATTTACAGTGGCACATTAGGTCCC | GCAGAGATACCAGCAGAGATACACG |
